# Linked-read based analysis of the medulloblastoma genome

**DOI:** 10.3389/fonc.2023.1221611

**Published:** 2023-07-28

**Authors:** Melissa Zwaig, Michael J. Johnston, John J.Y. Lee, Hamza Farooq, Marco Gallo, Nada Jabado, Michael D. Taylor, Jiannis Ragoussis

**Affiliations:** ^1^ Victor Phillip Dahdaleh Institute of Genomic Medicine and Department of Human Genetics, McGill University, Montreal, QC, Canada; ^2^ Alberta Children’s Hospital Research Institute, Arnie Charbonneau Cancer Institute, and Department of Biochemistry and Molecular Biology, Cumming School of Medicine, University of Calgary, Calgary, AB, Canada; ^3^ Department of Pathology and Center for Cancer Research, Massachusetts General Hospital and Harvard Medical School, Boston, MA, United States; ^4^ Broad Institute of Harvard and Massachusetts Institute of Technology (MIT), Cambridge, MA, United States; ^5^ BioBox Analytics Inc., Toronto, ON, Canada; ^6^ Department of Human Genetics, McGill University, Montreal, QC, Canada; ^7^ The Research Institute of the McGill University Health Centre, Montreal, QC, Canada; ^8^ Department of Pediatrics, McGill University, Montreal, QC, Canada; ^9^ Division of Neurosurgery, The Arthur and Sonia Labatt Brain Tumour Research Centre and the Developmental and Stem Cell Biology Program, The Hospital for Sick Children, Toronto, ON, Canada; ^10^ Texas Children’s Cancer Center , Hematology-Oncology Section and Department of Pediatrics – Hematology/Oncology and Neurosurgery, Baylor College of Medicine, Houston, TX, United States

**Keywords:** medulloblastoma, linked-reads, enhancer hijacking, extrachromosomal DNA, whole-genome sequencing, RNA sequencing

## Abstract

**Introduction:**

Medulloblastoma is the most common type of malignant pediatric brain tumor with group 4 medulloblastomas (G4 MBs) accounting for 40% of cases. However, the molecular mechanisms that underlie this subgroup are still poorly understood. Point mutations are detected in a large number of genes at low incidence per gene while the detection of complex structural variants in recurrently affected genes typically requires the application of long-read technologies.

**Methods:**

Here, we applied linked-read sequencing, which combines the long-range genome information of long-read sequencing with the high base pair accuracy of short read sequencing and very low sample input requirements.

**Results:**

We demonstrate the detection of complex structural variants and point mutations in these tumors, and, for the first time, the detection of extrachromosomal DNA (ecDNA) with linked-reads. We provide further evidence for the high heterogeneity of somatic mutations in G4 MBs and add new complex events associated with it.

**Discussion:**

We detected several enhancer-hijacking events, an ecDNA containing the *MYCN* gene, and rare structural rearrangements, such a chromothripsis in a G4 medulloblastoma, chromoplexy involving 8 different chromosomes, a *TERT* gene rearrangement, and a *PRDM6* duplication.

## 1 Introduction

Medulloblastoma (MB) is the most common malignant pediatric brain tumor with an incidence of 0.16-0.53 per 100,000 population, with children 0-9 years having the highest incidence (1). MBs are split into four molecularly distinct subgroups each with their own prognosis, expression, epigenetic, and mutational profiles (2). The groups are *wingless* medulloblastomas (WNT-MB), *sonic-hedgehog* medulloblastomas (SHH-MB), Group 3 medulloblastomas (G3-MB), and Group 4 medulloblastomas (G4-MB). In children, WNT-MB have the best prognosis of any MB subtype (3). They are characterized by activation of the WNT pathway mainly by means of mutations in *CTNNB1* and a recurrent complete or partial monosomy of chromosome 6 (4, 5; 3). A subset of WNT-MBs are caused by germline *APC* mutations which causes a predisposition to MB (3). SHH-MBs are characterized by the activation of the SHH pathway with the most commonly affected genes being *PTCH1*, *SUFU*, *SMO*, *GLI1*, *GLI2* and *MYCN* (6) as well as mutations in the *TERT* promoter (7), *TP53* and *PTEN* (8). Recently, a non-coding mutation in the U1 spliceosomal small nuclear RNAs (snRNAs) which was found to occur in 50% of SHH-MBs and leads to the inactivation of *PTCH1* and activation of *GLI1* and *GLI2* (9).

Until recently, the molecular mechanisms that differentiated group 3 and group 4 medulloblastomas were poorly understood since many genes were mutated in both subtypes (2, 3). In G4-MBs in particular, recurrent mutations were detected in a plethora of different driver genes but only in a small subset of tumors (2, 3). However, recent work by Hendrikse et al. has shown that most of the genes mutated in G4-MBs are either part of or interact with the core binding factor alpha (CBFA) complex which they suggest is required for the normal development of the rhombic lip (RL) into the ventricular zone (VZ) and sub-ventricular zone (SVZ) (10). These genes include *CBFA2T2, CBFA2T3, RUNX1T1*, *KDM6A*, and *KDM2B*, which are typically mutated or deleted, and *GFI1*, *GFI1B, PRDM6*, and *OTX2*, which are recurrently overexpressed. Three of the upregulated genes are affected by structural variants (such as deletions, duplications and inversions) that lead to the enhancer hijacking and overexpression of *GFI1*, *GFI1B* and *PRDM6* (via *SNCAIP* amplification) (11, 12).

Gain of the 17q and loss of 17p (termed isochromosome 17q) is also recurrently found in both G3-MBs and G4-MBs (13) as well as loss of chromosomes 8, 11p and X, and gain of chromosomes 7 and 18q in G4s (14). Additionally, *MYCN* is found amplified in 5-6% of G3 and G4 tumours while *MYC* amplification are found exclusively in G3 tumors (about 17% of cases) (12). *TERT* mutations are also found in all MB subtypes with the exception of WNT-MB although they occur at the highest rate in SHH-MBs (12). Oncogene amplification occurs in all MBs (except WNT-MB) by means of extrachromosomal DNA (ecDNA) with *MYCN* and *MYC* being the genes most commonly involved across all subtypes (15).

Structural variants (SVs) and their breakpoints can be difficult to detect using short-read Illumina sequencing since the read length is much smaller than the variants of interest. Long-read sequencing with Oxford Nanopore Technology (ONT) or PacBio (PB) is proving itself as an effective tool to identify structural variants in both normal and cancer genomes (16–20), however, long-read technologies are still costly and require much more high molecular weight (HMW) DNA input (at least 1.5µg). Linked-read sequencing has also been shown to be effective in identifying complex structural rearrangements, including complex events such as chromothripsis (21–24) as well as point mutations (25). It combines long-range genome information with the accuracy of short-read Illumina sequencing while requiring only low DNA input amounts (1-10ng). This low input requirement allows the method to be applied in samples where DNA quantity is limited and costs are comparable to standard WGS with Illumina. Although 10x Genomics has discontinued their linked-read technology (10X-LR), alternatives have been developed by Illumina (Complete Long Read sequencing), MGI (stLFR) (26), Universal Sequencing Technology (TELL-Seq) (27) and others (28).

This paper aims to perform a comprehensive analysis of medulloblastoma genomes by characterizing single nucleotide variants (SNVs) as well as rare driver events caused by large SVs using 10x Genomics linked reads. Additional validation and integration was done using short-read WGS, RNA-Seq and long-read Nanopore and PacBio sequencing. We also show for the first time that 10X-LR can be used for the detection of ecDNAs as validated by Hi-C. In order to explore the application of alternative linked-read technologies, we generated TELL-Seq (27) libraries for 4 of the tumor samples and validated the somatic SVs detected by 10X-LR. Using these datasets, we aim to expand the understanding of medulloblastoma biology by identifying previously uncharacterized structural variation. Identification of SVs can be used to guide diagnosis, personalize the selection of chemotherapies and monitor patient response to treatment (12) highlighting the importance of developing highly sensitive, low-cost genomic assays which could eventually be used in routine clinical practice.

## 2 Results

We generated 10X-LR tumor and normal data for 25 patients (21 G4-MB, 2 G3-MBs and 2 SHH MBs) in order to detect complex structural events driving tumorigenesis (**Table 1**; **Supplemental Table 1**; **Additional Table 1**). Of these 25 samples, 13 were previously characterized by WGS (12) which we reanalyzed using our high-sensitivity pipeline. RNA-Seq data was also produced for 13 samples and used for validation of enhancer hijacking events and expression of somatic SNVs.

**Table 1:** Sample table with known variants.

Sample	Age	Sex	Diagnosis	Characterized with WGS by Northcott et al.	Known Variants
MDT-AP-0074	3.29	M	Group 4	Yes	Enhancer hijacking of *PRDM6* Germline *BRCA2* (p.Tyr3225IlefsTer30, mostly lost in tumor)
MDT-AP-1206	1	F	Group 4	Yes	Enhancer hijacking of *GFI1B*
MDT-AP-1209	8	M	Group 4	Yes	*CDK6* (AMP of 7q21.2)
MDT-AP-1367	8.13	M	Group 4	Yes	
MDT-AP-1405	8.79	M	Group 4	Yes	Germline *RAD51D* (p.Asp98ValfsTer25)
MDT-AP-2075	7	F	Group 4	Yes	
MDT-AP-2078	5.4	F	Group 4	No	
MDT-AP-2130	9	M	Group 4	Yes	*TERT* promoter SNV (C228T)
MDT-AP-2151	13	M	Group 4	Yes	
MDT-AP-2407	6	M	Group 4	Yes	
MDT-AP-2638	NA	M	Group 4	No	
MDT-AP-2673	10.1	F	Group 4	Yes	Enhancer Hijacking of *GFI1B*
MDT-AP-2849	10	F	Group 4	Yes	Germline *ATM* (p.Arg2136Ter​)
MDT-AP-2857	12	M	Group 4	No	
MDT-AP-2859	17	M	Group 4	Yes	
MDT-AP-2878	9	M	Group 4	Yes	Possible Enhancer Hijacking of *GFI1* but no RNA for validation Focal AMP of *CDK6*
MDT-AP-2940	22	F	Group 4	No	
MDT-AP-3670	7	M	Group 4	No	
MDT-AP-3716	15	F	Group 4	No	
MDT-AP-3743	6	M	Group 4	No	
MDT-AP-3769	3	F	Group 4	No	
MDT-AP-3667	11.3	F	Group 3	No	
MDT-AP-4037	9.5	M	Group 3	No	
MDT-AP-3724	14	F	SHH	No	
MDT-AP-3862	2	M	SHH	No	

### 2.1 Structural variants and point mutations in G4 medulloblastomas

**Figure 1:**
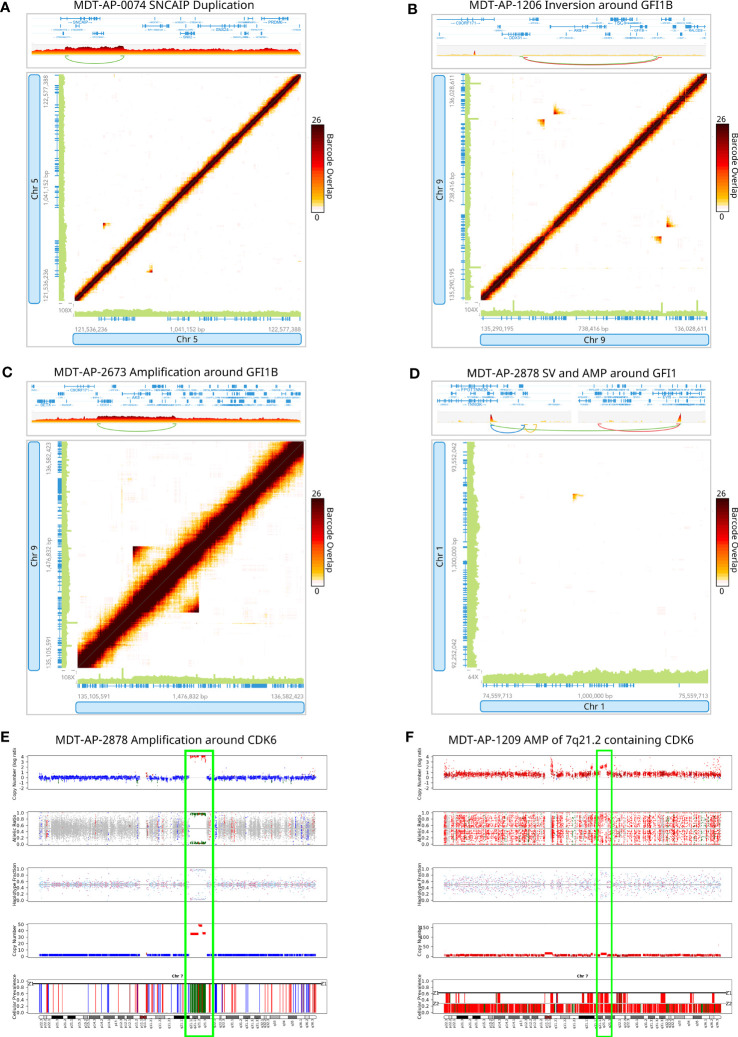
Detection of structural variants around recurrently mutated genes. 10X-LR data supporting **(A)** a *SNCAIP* duplication in MDT-AP-0074, **(B)** an inversion around *GFI1B* in MDT-AP-1206, **(C)** an amplification around *GFI1B* in MDT-AP-2673, **(D)** an structural variant and amplification around *GFI1* in MDT-AP-2878, and **(E)** an amplification around *CDK6* in MDT-AP-2878, visualization of the barcode overlap shown as heat maps in Loupe. Axes represent genomic regions and the colour of the points represents the number of barcodes that map to both of these regions. **(F)** Copy number profile of chromosome 7 showing the amplification of 7q21.1, which contains *CDK6*, in MDT-AP-1209, calculated and plotted with TitanCNA.

**Figure 2:**
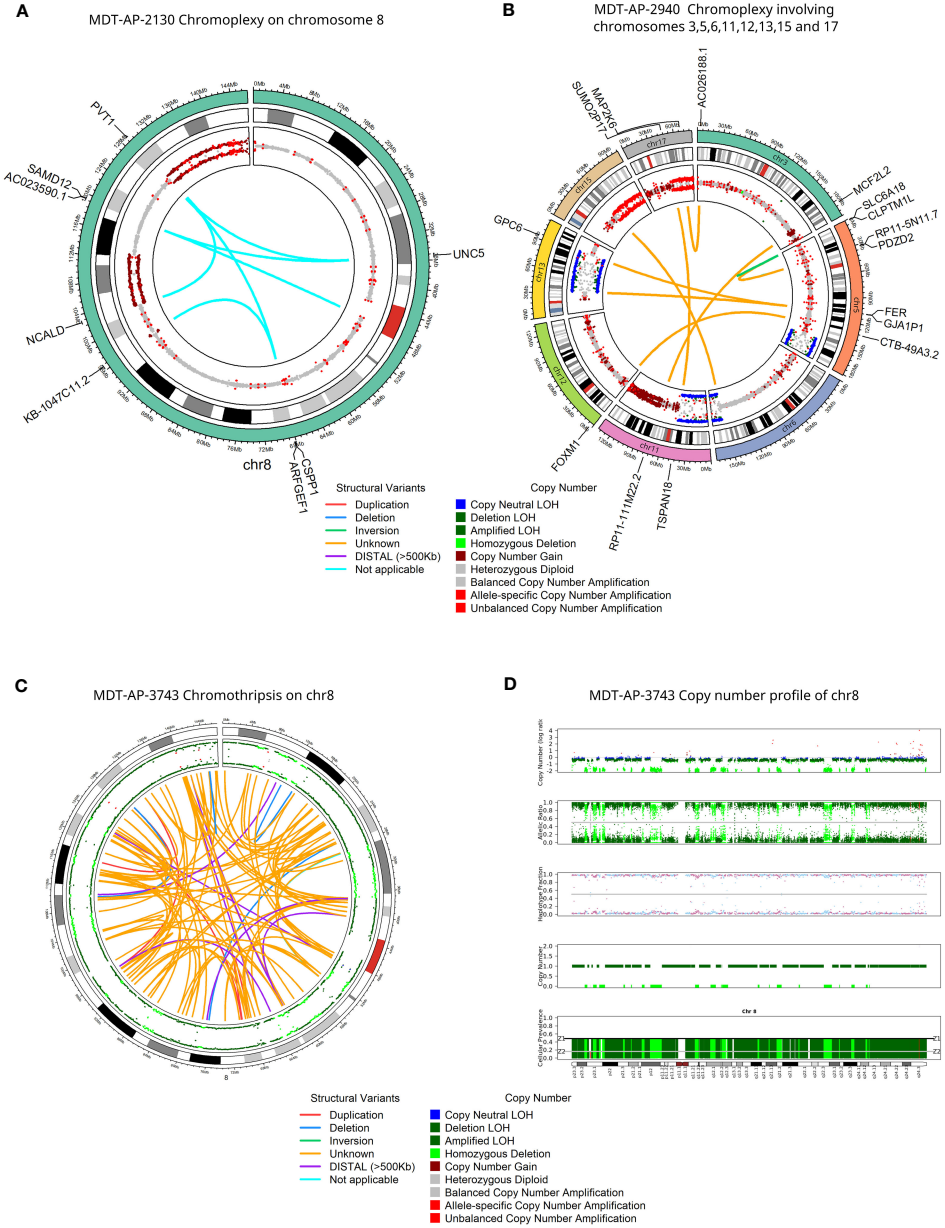
Detection of rare complex structural variants in G4 medulloblastomas. Circos plots for 10X-LR datasets showing **(A)** chromoplexy on chromosome 8 in MDT-AP-2130, **(B)** chromoplexy involving chromosomes 3, 5, 6, 11, 12, 13, 15 and 17 in MDT-AP-2940, and **(C)** chromothripsis on chromosome 8 in MDT-AP-3743. Outer circle shows allele frequency, as calculated by TitanCNA, were colour indicates the type of copy number change relative to the normal sample. Inner circle shows manually confirmed somatic SVs detected by 10X-LR and/or WGS and/or ONT and/or PacBio, colour indicates the type of SV **(D)** Copy number profile of chromosome 8 showing chromothripsis in MDT-AP-3743, calculated and plotted with TitanCNA.

In first instance, we generated tumor-normal 10X-LR datasets for the 13 samples that were part of the Northcott et al. study, and analyzed them using our in-house 10X-LR pipeline (see *Methods*) to identify previously undiscovered structural variants and provide a comprehensive list of somatic structural rearrangements. In addition, we re-analyzed the existing WGS data using an enhanced SV detection pipeline which uses 6 different SV callers in order to improve sensitivity (see *Methods*) (29). Combining the results from our WGS and 10X linked-read pipelines, we detected 265 somatic SV (which were manually confirmed using Loupe) in the 13 samples previously characterized by WGS (**Additional Table 2**, contains breakpoint coordinates for each technology and caller). Of these, 74 were detected by both 10X-LR and WGS, 173 were found by 10X only, and 18 were called by WGS only but visually confirmed in the 10X data. Our findings include mutations in recurrently mutated genes such as a *SNCAIP* duplication (**Figure 1A**), an inversion and an amplification in *GFI1B* (**Figures 1B, C**), an intrachromosomal rearrangement in *GFI1* (**Figure 1D**), and 2 large amplifications which include the *CDK6* locus (MDT-AP-2878, chr7: 86,891,518- 95,624,126, **Figure 1E**, MDT-AP-1209, chr7:90,074,594-93,426,132, **Figure 1F**) all of which were previously detected by Northcott et al. and validated by 10X-LR (12). We also detected a novel complex rearrangement on chromosome 8 in MDT-AP-2130 (validated by WGS, **Figure 2A**). Additionally, we detected a complex event in MDT-AP-2878 involving chromosomes 2 and 16 with a breakpoint downstream of *IDH1* that had not been previously characterized but was validated in the re-analyzed WGS data (**Supplemental Figures 1A, B**).

Next, we applied 10x Genomics linked-read sequencing in 12 uncharacterized samples: 8 new G4 medulloblastomas, two G3 and two SHH medulloblastomas (see *Findings in non-G4 medulloblastomas* below). In the 8 G4 medulloblastomas that had not been characterized previously, we detected 147 somatic SVs that were manually confirmed using Loupe (**Additional Table 2**). MDT-AP-2940 was found to have chromoplexy involving chromosomes 3, 5, 6, 11, 12, 13, 15 and 17 (**Figure 2B**) as well as a complex event on chr5 leading to the amplification of *TERT* (**Supplemental Figure 1C**). Additionally, we detected chromothripsis in one sample involving chromosome 8 co-occurring with loss of 17p which contains *TP53* (MDT-AP-3743, **Figures 2C, D**). Loss of *TP53* is thought to be required for chromothripsis and although 17p loss in common in G4s, chromothripsis is rare (30).

**Figure 3:**
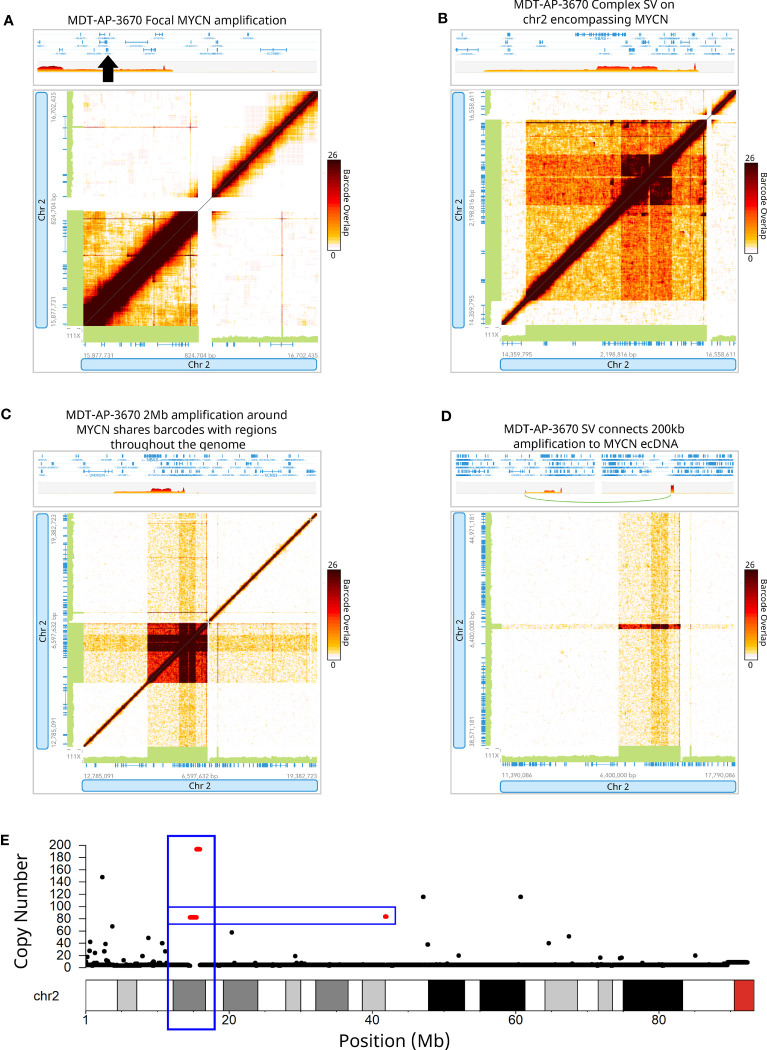
Detection of extrachromosomal DNA using linked-reads. 10X-LR data supporting **(A)** a duplication of *MYCN*, **(B)** a complex SV on chromosome 2 encompassing *MYCN*, **(C)** 2Mb amplification around *MYCN* shares barcodes with regions throughout the genome indicating ecDNA, **(D)** an SV which connects a 200kb amplification at 42Mb to the *MYCN* ecDNA, visualization of the barcode overlap shown as heat maps in Loupe. Axes represent genomic regions and the colour of the points represents the number of barcodes that map to both of these regions. **(E)** Copy number profile of chromosome 2 showing the amplification of the *MYCN* region (chr2:15Mb) and upstream region (chr2:42Mb), calculated and plotted with TitanCNA.

*MYCN* was found amplified in MDT-AP-3670 and further analysis showed that this was part of a much larger complex SV and amplification with a breakpoint connecting it to a region 27.4Mb downstream on chromosome 2 (**Figures 3A–D**). Interestingly, both of these events were shown to share barcodes across the genome which suggests that there are many copies of these regions within the nucleus that are being caught within the emulsion created by the 10X-LR protocol (**Figures 3C, D**). We hypothesized that this patterning indicated ecDNA which we then validated using Hi-C data from the same sample (**Supplementary Figure 2**). Hi-C has previously been shown to be able to detect ecDNAs in a wide-range of tumors and cell lines (31–34). Additionally, copy number calls generated from 10X-LR using TitanCNA indicated approximately 75 copies of chromosome 2 from 14.6-16.3Mb and 41.7-41.9Mb as well as even higher amplification (~125 copies) of chromosome 2 from 15.5-15.74Mb and 15.75-15.96Mb which contains additional rearrangements (**Figure 3E**). As far as we are aware, this represent the first time ecDNA has been identified using 10X linked-reads.

In terms of point mutations, SNVs described as functional SNVs and indels by Northcott et al. were manually validated in the linked-read data and as well as in the RNA-Seq where applicable (**Additional Table 3**) (12). These include a *TERT* promotor mutation in MDT-AP-2130 (C228T, **Supplemental Figure 1D**) as well as germline mutations in *BRCA2* (p.Tyr3225IlefsTer30, Pathogenic/Likely pathogenic in ClinVar), *RAD51D* (p.Asp98ValfsTer25, likely pathogenic in ClinVar) and *ATM* (p.Arg2136Ter, Pathogenic/Likely pathogenic in ClinVar), all of which are associated with the double-stranded break repair pathway and cancer predisposition syndromes (35). Additionally, analysis of the linked-red data allowed us to detect two mutations in *KDM6A*, a frameshift variants in *KMT2D* [known to be recurrently mutated in G4-MB (10)], a mutation in *CREBBP* annotated as likely pathogenic in ClinVar, and a second *TERT* promoter mutation (C228T).*KDM6A* is a lysine demethylase recurrently mutated in both G3 and G4 medulloblastomas (36). The mutations were a missense mutation (p.R1255W, MDT-AP-2151, validated in WGS) previously detected in carcinomas of the pancreas, endometrium, prostate, breast, and skin as well as a truncating mutation annotated as likely pathogenic in ClinVar (R1331fs, MDT-AP-2857) found in the germline of three patients with Kabuki syndrome 2 (https://www.ncbi.nlm.nih.gov/clinvar/variation/216950/).

### 2.2 Enhancer hijacking in G4 medulloblastomas

**Figure 4:**
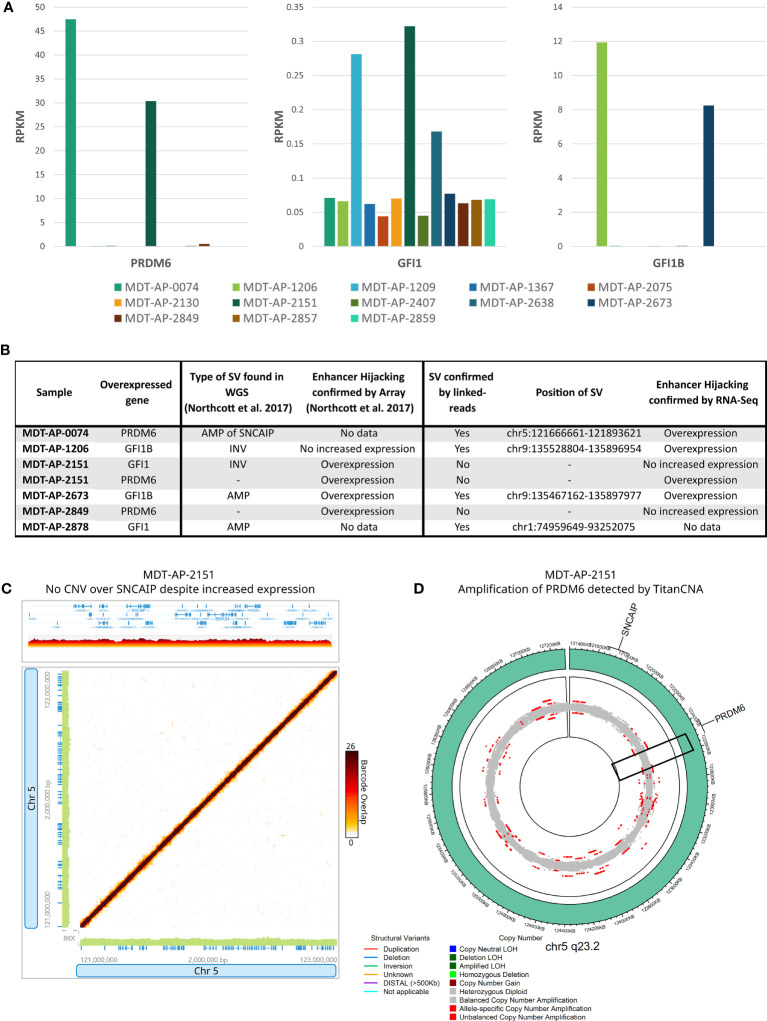
Enhancer hijacking in G4 medulloblastomas. **(A)** Bar graphs showing expression of *PRDM6, GFI1* and *GFI1B* in all samples with RNA-Seq data available. **(B)** Table showing cases of enhancer hijacking in terms of SV calls and expression as described in Northcott et al. and this paper. **(C)** 10X-LR data showing no CNV over *SNCAIP* or *PRDM6* in MDT-AP-2151 despite increased RNA-Seq expression, visualization of the barcode overlap shown as heat maps in Loupe. Axes represent genomic regions and the colour of the points represents the number of barcodes that map to both of these regions. **(D)** Circos plots for 5q23.3 showing a duplication of *PRDM6* in MDT-AP-2151 as allele frequency, as calculated by TitanCNA, were colour indicates the type of copy number change relative to the normal sample.

In the G4 medulloblastomas, SVs affecting *GFI1* and *GFI1B* and the recurrent tandem duplication of *SNCAIP* are known to cause overexpression of *GFI1*, *GFI1B* and *PRDM6*, respectively, by putting them under the control of super-enhancer regions (termed enhancer hijacking, EH). We used the RNA-Seq data, which was available for 13 samples, to validate these EH events. Expression levels supported enhancer hijacking of *GFI1B* in MDT-AP-1206 and MDT-AP-2673 as well as *PRDM6* in MDT-AP-0074 (**Figures 4A, B**). Interestingly, MDT-AP-2151 was also shown to have overexpression of *PRDM6* despite no evidence of a tandem duplication of *SNCAIP* by either Northcott et al. or us (12) (**Figure 4C**). However, copy number data from TitanCNA suggests a small duplication over *PRDM6* which explains the increased expression and suggests that tandem duplication of *SNCAIP* may not be the only mechanism leading to overexpression of *PRDM6* (**Figure 4D**).

### 2.3 Copy-number variants in G4 medulloblastomas

Group 4 medulloblastomas are also known to be tetraploid, with 11/21 samples in this study having a ploidy of 4 (52%, **Supplemental Table 1**). Group 4 MBs are also characterized by extensive copy number variants, two of the most characteristic being gain of chromosome 17q (14/21, 66%) with or without loss of chromosome 17p (13/21, 62%, contains *TP53*), as well as a gain of chromosome 7 (11/21, 52%) and loss of chromosome 8 (10/21, 47%) (14) (**Supplemental Figure 3**).

### 2.4 Findings in non-G4 medulloblastomas

We detected 11 manually confirmed somatic variants in the previously uncharacterized non-G4 medulloblastomas (2 in 2 SHH-MBs and 9 in 2 G3-MBs, **Additional Table 2**). One SHH tumor was found to have a *TERT* promoter mutation (C228T, MDT-AP-3724) as well as a previously described *CREBBP* mutation (p.R1446L c.4337G>T), a single-base deletion in exon 34 of lysine-specific methyltransferase 2D (*KMT2D*), an interchromosomal translocation between chromosomes 3 and 14 (**Supplemental Figure 4A**), 4 copies of 3q and loss of 14q24.1-q32.33 (**Supplemental Figure 4B**). The other was characterized by an interchromosomal translocation between chromosomes 7 and 18 (MDT-AP-3862, **Supplemental Figure 4C**), a gain of 7q31.2-36.3, loss of 20 and loss of homozygosity on 10q which contains *SUFU* (**Supplemental Figure 4D**).

The group 3 medulloblastomas were mainly characterized by copy number changes and structural variants although none were recurrent between the two samples (**Additional Table 2**). Of note, one G3-MB was found to have a germline interchromosomal translocation between chromosomes 2 and 5 occurring near 2 protocadherin genes and a protocadherin gene cluster (MDT-AP-4037, **Supplemental Figures 4E, F**). Previous work suggests that protocadherins may play a role in tumorigenesis in medulloblastomas (37, 38).

### 2.5 Cross-validation using long-read PacBio and Oxford Nanopore data

We generated PacBio data from 5 G4 MB tumor-normal pairs where additional DNA material was available (7-19X coverage, **Supplemental Table 1**). In addition, we were able to generate paired tumor-normal Nanopore data from two of these G4 tumor samples at 15-30X coverage plus deep sequencing data from the tumor of MDT-AP-2673 (53x coverage). All samples with long-read data also had WGS and RNA-Seq data available (**Supplemental Table 1**). Analysis of the long-read data allowed the detection of 16 somatic SVs that had been confirmed as somatic and included the focal events around *GFI1B* and *SNCAIP* leading to enhancer hijacking (**Additional Table 2**). Additionally, we detected 4 SVs found uniquely by long-reads which we validated as somatic (**Additional Table 2**).

### 2.6 Comparison of linked-read technologies

Since 10x Genomics has discontinued their linked-read kit, we decided to test the TELL-Seq library kit by generating data for 4 tumor samples in order to compare the SV calls. We chose samples which had somatic SVs in *GFI1B* (MDT-AP-1206 and MDT-AP-2673), in *TERT* (MDT-AP-2940) and *GFI1* (MDT-AP-2878) that had previously been detected by 10X-LR.QC metrics for both technologies were generated using the LongRanger pipeline. On average, the 10X-LR samples had longer mean molecules lengths compared to TELL-Seq although this is likely due to sample degradation over time since the same HMW DNA extractions we used to generate both tumor linked-read datasets about 2 years apart (**Supplemental Table 2**). As a result, the 10X-LR data out-performed TELL-Seq in terms of the number of phased SNPs and longest phase block. Both technologies had similar numbers of large SV and short deletions calls made by LongRanger (**Supplemental Table 2** and **Additional Table 1**); however, the TELL-Seq data had much more even coverage compared to the 10X-LR data (**Supplemental Figure 5**).

**Figure 5:**
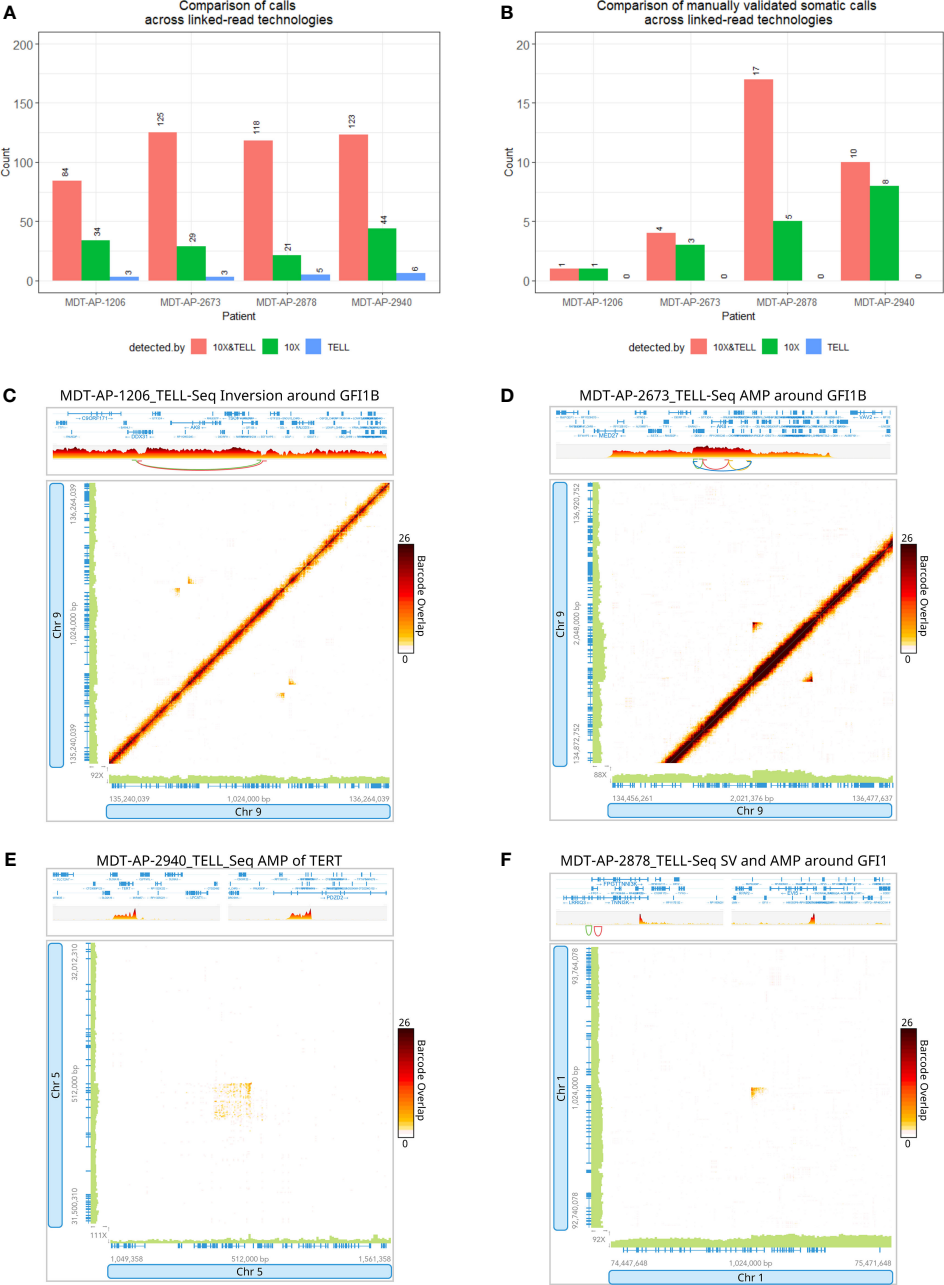
Detection of variants with 10x Genomics and Universal Sequencing Technologies’ linked-read protocols. Bar graphs comparing **(A)** the number of SV calls and **(B)** the number of manually validated SV calls detected by both 10X-LR and TELL-Seq, 10X-LR only and TELL-Seq only. TELL-Seq data supporting **(C)** an inversion around *GFI1B* in MDT-AP-1206, **(D)** an amplification around *GFI1B* in MDT-AP-2673 and, **(E)** a structural variant and amplification around *GFI1* in MDT-AP-2878, and **(F)** the amplification of *TERT* in MDT-AP-2940, visualization of the barcode overlap shown as heat maps in Loupe after conversion of TELL-Seq data to LongRanger format. Axes represent genomic regions and the colour of the points represents the number of barcodes that map to both of these regions.

Nearly all high-quality somatic calls (detect by at least 2 callers and >10kb) made in the 10X-LR data were validated by TELL-Seq. 84-125 calls were made by both technologies, 21-44 calls were made by 10X only, and 3-6 were made by TELL-Seq only (**Figure 5A** and **Additional Table 4**). 63 somatic calls were made across the 4 samples of which 49 were called by at least 2 callers in the 10X-LR dataset (the rest where either detected by WGS or a single caller in the 10X-LR datasets). Of these 49 calls, 32 were detected in both the 10X-LR and TELL-Seq datasets from the same patient (**Figure 5B**). Of the 22 somatic SVs which occurred in a gene of interest or are part of a complex genomic event such as chromoplexy, 14 were detected in both the 10X-LR and TELL-Seq datasets (**Additional Table 4**) and included both enhancer hijacking events in *GFI1B* (**Figures 5C, D**) and the amplification around *TERT* (**Figure 5E**). Eight somatic SVs were only detected in the 10X-LR, however, manual inspection of the TELL-Seq data in Loupe allowed us to confirm visually the presence of the SVs not called by TELL-Seq including the SV affecting *GFI1* in MDT-AP-2878 (**Figure 5F**).

## 3 Discussion

In this paper, we applied 10x Genomics linked-read data to 25 medulloblastomas in order to identify additional rearrangements in 13 samples previously characterized by WGS and establish cross validation of findings. Using our custom 10X-LR analysis pipeline, we were able to detect 96 SVs not previously described in these samples, of which 86 could be validated when using our high-sensitivity WGS pipeline. Additionally, we characterized 12 new samples in which we detected 158 manually confirmed somatic SV including a *TERT* promoter mutation and complex SV involving the *TERT* gene, chromoplexy involving 8 chromosomes, chromothripsis involving chromosome 8, ecDNA amplification of *MYCN* and a germline interchromosomal SV occurring near a medulloblastoma candidate gene family. A summary of all variants of interest identified in our datasets can be found in **Table 2** and all SV calls that were manually validated as somatic across all technologies and callers can be found in **Additional Table 2**.

**Table 2:** New and cross-validated somatic variants.

Sample	Diagnosis	Estimated ploidy	Chromosomal CNVs	Variants of Interest
MDT-AP-0074	Group 4	4	Gain (≥5 copies) of 2p25.3-p16.1, 4p16.3-p15.31, 7, 12q24.21-q24.33, 15q21.3-q26.3, 17q12-q25.3 LOH of 2q37.1-q37.3, 8p23.3-p23.1, 10q22.3-q26.3, 13q12.13-q21.1, 22q13.2-q13.33	*Enhancer hijacking of *PRDM6* *Germline *BRCA2* (p.Tyr3225IlefsTer30, mostly lost in tumor) Interchr SV between chr2&12, chr2&8, chr4&10, and chr17&22
MDT-AP-1206	Group 4	4	No large CNVs	*Enhancer hijacking of *GFI1B* *FLG (p.H2268R) *PDE4DIP (NM_001278267.1)
MDT-AP-1209	Group 4	4	Gain (≥5 copies) of 7, 17q 3 copies of 1, 3p22.1-q35.3, 10q, 11, 17p LOH of 8 (2 copies of the same haplotype)	*CDK6 (AMP of 7q21.2) *PLXNA2 (p.E1675K) Interchr SV between chr10&12
MDT-AP-1367	Group 4	4	Gain (≥5 copies) of 7, 18 3 copies of 4, 5, 10, 19q 2 copies of 1, 3, 13, 16p, 19p, 20, 21, 22 1 copy of 8, 11p, 16q	*COL1A1 (p.D168G) *ITGA2 (p.D877A) *PCLO (p.R4078H) *SMARCA4 (p.G1068S) Interchr SV between chr11&16
MDT-AP-1405	Group 4	2	Gain of 7q, 12q23.2-24.33, 17q Loss of 3p21.3-p14.3, 5p15.33-p14.3, 5q33.1-q35.3, 8, 17p	*Germline *RAD51D* (p.Asp98ValfsTer25) *SPTBN2 (p.N329S) Interchr SV between chr5&7 and chr5&12
MDT-AP-2075	Group 4	4	Gain of 17q Loss of 17p High number of small gains and losses	*SLIT2 (p.E1494X) Interchr SV between chr12&18
MDT-AP-2078	Group 4	4	Gain (≥5 copies) of 2p25.3-q14.3, 4, 6, 7, 9, 17, 18, 20 3 copies of 2q21.1-q37.3 1 copy of 8p21.2-q21.3 LOH of 3p21.31-q29, 8p23.3-q21.3, 10 (2 copies of the same haplotype)	
MDT-AP-2130	Group 4	2	Gain (≥3 copies) of 6q14.3-q27, 17 1 copy of 8q22.3-q23.2, 8q24.22-q24.3, 10	Somatic SVs on chr8 *TERT promoter SNV (C228T) Interchr SV between chr6&X
MDT-AP-2151	Group 4	2	Gain of 17q Loss of 17p High number of small gains and losses	*KDM6A* (p.R1255W) *Overexpression of *PRDM6*, AMP of *PRDM6* validated by TitanCNA and RNA-Seq *MUC17 (p.M1807T) *MUC17 (p.T1808N) *ZIC1 (p.P301S)
MDT-AP-2407	Group 4	2	Gain of 1q42.12-q44, 7, 17q Loss of 8p23.3-p12, 17p	Interchr SV between chr1&8 and 12&19
MDT-AP-2638	Group 4	4	Gain (≥5 copies) 11q13.2-q24.1, 12q24.13-q24.33, 17q LOH of 12p13.2-q13.1, 13q21.23-q31.1,16q, 17p	
MDT-AP-2673	Group 4	4	Gain (≥5 copies) of 7, 17q 3 copies of 11, 12p13.33-p12.1, 13, 19, 20 1 copy of 8p23.3-p22.1, 18q22.1 LOH copy of 8q22.2-q24.3, 17p	*Enhancer Hijacking of *GFI1B* *MDN1 (p.S3987X) Interchr SV between chr2&13 and chr8&12
MDT-AP-2849	Group 4	2	Gain of 1q44, 2p25.3-p22.2, 7q, 12p13.33-p12.1, 12q23.3-q24.33 Loss of 2q13-q24.1, 2q37.3, 5q32-q35.3, 9p24.3-p24.1, 11q23.3-q25, 16q21-q24.3 LOH of 2q24.2-q37.2	*Germline *ATM* (p.Arg2136Ter​) *H1FNT (p.A150T) *KBTBD4 (InDel, p.G292delinsGEG) *VWDE (p.H1211N) Interchr SV between chr1&5, chr2&9, chr2&11, chr2&12
MDT-AP-2857	Group 4	4	Gain of 17q Loss of 17p, 19 High number of small gains and losses	*KDM6A (p.R1331fs)
MDT-AP-2859	Group 4	4	Gain (≥5 copies) of 4, 5, 6, 7, 16, 17q, 20, 21 3 copies of 2, 8 1 copy of 17q	*ARID1B (p.N1456S) *MUC16 (p.W1384C)
MDT-AP-2878	Group 4	2	Gain of 1p31.1-p22.2, 2q23.3-q24.2, 2q34-q35, 7p21.12-21.3, 8p23.1-21.2, 16q23.2-q24.3, 17q22-q25.3, 18q Loss of 8q21.12-24.3	Complex event involving chromosomes 2 and 16 with breakpoint near *IDH1* *Possible Enhancer Hijacking of *GFI1* but no RNA for validation *Focal AMP of *CDK6* *ICOSLG (SNV, p.A272V) *KBTBD4 (InDel, p.R296delinsHR *PKHD1L1 (InDel, p.41934194del) Interchr SV between chr8&17
MDT-AP-2940	Group 4	4	Gain of 7, 8q23.3-24.3 (with LOH), 11q, 15q21.1-26.3, 16, 17q21.3-25.3 Loss of 5q33.1-35.3, 6q25.3-27, 8p23.3-21.3, 8q11.1-24.3, 11p, 13q13.1-31.3	Complex event on chr5 involving *TERT* Chromoplexy of chromosomes 3,5,6,11,12,13,15 and 17
MDT-AP-3670	Group 4	4	Gain (≥5 copies) of 5, 7, 12p13.33-13.32, 17q, 18 3 copies of 3, 6q24.3-q27, 8, 10, 11, 20 LOH 13, 16q, 17p	ecDNA amplification of *MYCN* Interchr SV between chr12&16
MDT-AP-3716	Group 4	2	Gain of 11q, 16p13.3, 17q Loss of 17p, 22q13.2-13.33 LOH of 19p	
MDT-AP-3743	Group 4	2	Gain of 6, 7, 17q, 18, 19, 21 Chromothripsis on chr8 (oscillating between 1 and 0) Loss of 3, 10, 11, 17p LOH of 12	Chromothripsis on chr8
MDT-AP-3769	Group 4	4	3 copies of 3, 6, 7, 9, 10, 11, 12, 13, 16, 18, 20 2 copies of 8, 19 LOH of 17p	
MDT-AP-3667	Group 3	2	Gain of 1q, 4, 5, 6, 7, 8q23.1-q24.3, 12, 14, 17, 18 LOH of 2, 9p, 19, 21	
MDT-AP-4037	Group 3	2	4 copies of 5, 18 3 copies of 1, 6, 7, 9 1 copy of 3, 4, 8, 10, 15, 16, 21 LOH of 2, 11, 17	Germline interchr SV between chr 2&5, protocadherin gene cluster
MDT-AP-3724	SHH	2	Gain of 3q12.3-q29 Loss of 14q24.1-q32.33	*TERT* promoter SNV (C228T) *CREBBP* (p.R1446L) *KMT2D* (p.D3048fs) Interchr SV between chr3&14
MDT-AP-3862	SHH	2	Gain of 7q31.2-q36.3 Loss of 20 LOH of 10q22.2-q26.3	Interchr SV chr7&18

*denotes variants called by Nothcott et al. and cross-validated with 10X-LR.

Using linked-reads, we identified both rare and novel mutational events in G4 medulloblastomas. These mutations include chromothripsis in a G4-MB which is rare despite the high frequency of loss of *TP53* (via loss of 17p) which is believed to be a requirement for chromothripsis (30). We identified two point mutations in *KDM6A*, which had not previously been identified in medulloblastomas, and validated germline mutations in *BRCA2*, *RAD51D* and *ATM*, which are all involved in DNA repair of double-stranded breaks as well as hereditary cancer syndromes (35). Medulloblastomas have long since been associated with germline mutations in *APC*, *PTCH1*, *SUFU* and *TP53* (39) and more recently in *BRCA2* and *PALB2* (40). To date, germline *RAD51D* mutations have only been associated with increased risk of ovarian cancer and *ATM* germline mutations have primarily been shown to increased risk of breast cancer as well as case familial cases of ovarian, prostate, and pancreatic cancers. However, both *RAD51D* and *ATM* are involved in the homologous repair pathway that also includes medulloblastoma susceptibility genes *TP53*, *BRCA2*, and *PALB2* (40). Additionally, we detected a novel germline interchromosomal variant in a G3 medulloblastoma. Interestingly, the breakpoint for this translocation on chromosome 5 falls 120kb way from protocadherin 12 (*PCDH12*), 200kb away from protocadherin 1 (*PCDH1*), and 500kb away from the protocadherin gamma (*PCDHG*) gene cluster. While none of these genes have been specifically investigated, mutations in other protocadherins, *PCDH9* (38) and *PCDH10* (37) have been identified as potential drivers in medulloblastoma. Despite the identification of rare variants and new SVs in recurrently affected genes, no novel recurrently mutated genes could be identified which is unsurprising considering the modest size of our dataset.

Lastly, we showed for the first time that ecDNA can be identified using linked-reads alone. Due to the high number of copies circulating within the nucleus, ecDNAs are randomly captured within the emulsions created by the 10x Genomics linked-read protocol. This makes the amplified region appear to be found at low levels throughout the genome and generates a similar pattern to Hi-C data were the ecDNA is shown to interact with the entirety of the genome (31–34).

In this paper, we show that linked-reads provide detailed characterization of many types of variants including SNPs, SVs, CNVs, chromothripsis and ecDNAs while also providing phasing and breakpoint information. The minimal input required by linked-read technologies makes them an appealing option for clinical diagnosis particularly when tumors are small or occur in regions which are surgically inaccessible but can still be biopsied. Limitations to linked-read technologies include evenness of coverage and difficulty with repetitive regions. The 10x Genomics protocol uses a polymerase with stand displacement to generate barcoded amplicons during the isothermal incubation step, however this leads to uneven coverage compared to standard PCR-free WGS although this seems to be less of an issue with the TELL-Seq protocol (**Supplemental Figure 5**). Additionally, since linked-reads are a short-read based technology, repetitive regions larger than the length of a read are still difficult to align with precision. Long-reads are more likely to span the entirety of a low complexity region, making alignment less difficult. Other alternatives to both linked-reads and long-reads include Illumina’s new Complete Long Read (CLR) protocol which land-marks long DNA fragments before tagmenting them and sequencing them with their existing chemistry. The land-marking allows long DNA fragments to be fully reconstructed computationally as opposed to linked-read technologies where barcoded reads represent a sampling of a HMW DNA molecule.

In conclusion, our work provides further evidence for the high heterogeneity of variants seen across G4 medulloblastoma and adds new complex events including a new mechanism of *PRDM6* overexpression *via* gene duplication. G3 and G4 medulloblastomas have been shown to be driven by SVs across many different genes (39). Our group and others have shown that technologies that provide long-range information are required to characterize the full spectrum of SVs in medulloblastomas (41).

## 4 Methods

### 4.1 10x Genomics linked-reads

10x Genomics linked-read libraries were generated for 25 tumors and corresponding normal samples. HMW DNA was extracted from tumors using phenol chloroform extractions or the Nanobind tissue kit (PacBio, Menlo Park, California, United States, cat# SKU 102-302-100) while matching blood samples were extracted using the QiaAmpBlood Kit (Qiagen, Hilden, Germany) or the Nanobind tissue kit (PacBio, Menlo Park, California, United States, cat# SKU 102-302-100) (**Additional Table 1**). Size selection was done with the SRE and SRE XS kits (PacBio, Menlo Park, California, United States, cat# SKU 102-208-200 and SKU 102-208-300) as needed and dependent on the availability of DNA (**Additional Table 1**). Concentration was assessed by Qubit™ dsDNA BR Assay Kit (ThermoFisher Scientific, cat# Q32853) and size distribution was profiled using the Femto Pulse (Genomic DNA 165 kb Kit, 3 hours run, Agilent Technologies, Inc., Santa Clara, California, United States, cat# FP-1002-0275). Samples and library preparation were done following the Chromium™ Genome Reagent Kits v2 User Guide (10x Genomics, Pleasanton, California, United States). Libraries concentration was assessed by qPCR (Roche, Basel, Switzerland, KAPA Library Quantification Kits - Complete kit (Universal), cat# 07960140001) and the size distribution of the libraries was evaluated using the Caliper LabChip (DNA High Sensitivity assay, PerkinElmer, Inc., Waltham, Massachusetts, United States). Libraries were sequenced using 150PE Illumina reads, either on a single lane of HiSeqX or pooled on a NovaSeq S4 flowcell. Average molecule length, calculated by LongRanger, ranged from 19kb-85kb for tumor samples and 42kb-104kb for normal samples (**Additional Table 1**).

Data was analyzed as detailed in Zwaig et al. (42). In brief, alignment and variant calling was done using 10x Genomics’ LongRanger pipeline followed by additional SV calling with GROC-SV (43), NAIBR (44) and LinkedSV (45) and SvABA (46) and copy number calling with TitanCNA 10x workflow (23). A custom R script was used to find calls made by multiple callers and we manually confirmed all SV calls detected by at least 2 callers and over 10kb in Loupe which are listed with breakpoint information and gene annotation in **Additional Table 2**. SVs labeled as “variants of interest” in **Additional Table 2** include all SVs occurring in a genes known to be recurrently mutated in G4 medulloblastomas (*CDK6, GFI1, GFI1B, MYCN, SNCAIP/PRDM6*), those occurring in or near genes known to be recurrently mutated in other cancers types (*IDH1* and *TERT*), those near suspected medulloblastoma genes (procadherin genes), and complex somatic variants such as chromoplexy and chromothripsis). These variants of interest are discussed in more detail within the results section. Only 3 other genes were mutated in more than one patient; these include *ARFGEF1* and *KB-1047C11.2* which contain breakpoints associated with chromoplexy and chromothripsis on chromosome 8 in MDT-AP-2130 and MDT-AP-3743, respectively, and *STEAP2-AS1* which is found near *CDK6* and contains breakpoints in both samples with *CDK6* amplifications.

### 4.2 Whole-genome sequencing

WGS data was available through Northcott et al. (12) and processed using the GenPipes Tumor-Pair pipeline for SV calling (*-t sv*) and SNP calling (-*t ensemble*) (29). We also ran SvABA on the WGS data (46).

### 4.3 Nanopore sequencing

Two tumors and their matching normal samples (MDT‐AP‐1367 and MDT‐AP‐1405) were sequenced on the MinIon (Oxford Nanopore Technologies Limited, Oxford, United Kingdom). MDT-AP-2673 was sequencing on 2 PromethION flowcells (Oxford Nanopore Technologies Limited, Oxford, United Kingdom). Both the MinIon and PromethIon libraries used 2µg of HMW DNA as input. Nanopore data was aligned to genome build b37 with minimap2 (version 2.17) using parameter *-ax map-ont* (47). Structural variants were called SVIM (48) (parameters *–min_mapq 7 –min_sv_size 50 –max_sv_size 100000*), NanoVar (49) (version 1.3.9, parameters *–data_type ont –mincov 2 –minlen 50*), CuteSV (50) (version 1.0.11, parameters *–min_size 50 –max_size 100000 –min_support 2 –min_mapq 7 –max_cluster_bias_INS 100 –diff_ratio_merging_INS 0.3 –max_cluster_bias_DEL 100 –diff_ratio_merging_DEL 0.3*), and Sniffles2 (51) (version 2.0.6, parameters *–minsupport 2 –mapq 7 –minsvlen 50 –non-germline*).

### 4.4 PacBio sequencing

PacBio data was available for 5 tumors and their matching normal samples. Samples were normalized to a concentration of 125pM and sequenced with 4-hour movies. Data was aligned to genome build b37 using NGMLR (51) (version 0.2.7) and SVs were called using Sniffles (51) (version 1.0.10, parameters *–min_support 2 –min_length 30*).

### 4.5 TELL-Seq

TELL-Seq libraries were generated using the same HMW DNA aliquots as the 10X-LR libraries. 5ng of HMW DNA was used per library (as recommended by the UST TELL-Seq™ WGS Library Prep User Guide V8) and quantified by Qubit™ dsDNA HS Assay Kit (ThermoFisher Scientific, cat# Q32854). Final libraries were assessed by qPCR (KAPA Library Quantification Kits) and Caliper LabChip. Libraries were sequenced using 150PE Illumina reads on 1 lane of NovaSeq SP. QC and barcodes correction was done using the TELL-Read pipeline, and SNP calling was done using the TELL-Sort pipeline. We used the ust10x tools to convert the TELL-Seq data to 10X-LR format and ran our in-house pipeline detailed above with the exception of GROC-SV which did not run to completion on the TELL-Seq data.

### 4.6 RNA sequencing

Bulk RNA-Seq data was generated for 13 samples and analyzed using the GenPipes RNA-Seq pipeline (29). Overexpression of genes affected by enhancer hijacking was measured using the reads per kilobase of transcript, per million mapped reads (RPKM) calculated by the pipeline.

### 4.7 Hi-C

Hi-C data was available for MDT-AP-3670 (unpublished work). Detailed description of the library preparation protocol and analysis workflow can be found in (42).

### 4.8 Comparison of SV calls across genomic technologies (10X-LR, WGS, ONT, PacBio)

A custom R script was used to find SVs detected by multiple technologies (i.e. were both the start and end breakpoints fell within ±1000bp of each other) and we manually assessed all SV calls made by 2 or more callers and larger than 10kb in size using Loupe. All manually confirmed somatic structural variant calls can be found in **Additional Table 2**.

### 4.9 Comparison between 10X-LR and TELL-Seq

Evenness of coverage was compared using BVAtools depthofcoverage (parameters, *–gc–minMappingQuality 15 –minBaseQuality 15 –ommitN –maxDepth 1000 –binsize 50000–summaryCoverageThresholds 0,1,2,3,4,5,6,7,8,9,10,11,12,13,14,15,16,17,18,19,20,21,22,23,24,25,26,27,28,29,30,31,32,33,34,35,36,37,38,39,40*) and plotted using the karyoploteR package in R. A custom R script was used to compare SVs made by both 10X-LR and TELL-Seq (i.e. were both the start and end breakpoints fell within ±1000bp of each other). All SV calls made by 2 or more callers and large than 10kb in size were manually validated using Loupe. All structural variant calls across both linked-read technologies and all callers can be found in **Additional Table 4**.

## Data availability statement

The datasets presented in this study can be found in online repositories. The names of the repository/repositories and accession number(s) can be found below: https://ega-archive.org, EGAS00001007064, https://ega-archive.org, EGAS00001001953.

## Ethics statement

Protocols for this study were approved by the Research Ethicsand Review Board (REB) at the McGill University Health Centre(Project number: 2018-4511) and affiliated hospital ResearchInstitutes. Patient samples were collected with informed consentfrom all research participants or their delegates.

## Author contributions

MZ and JR contributed to the study conception and design. Generation and analysis of linked-read data, analysis of Nanopore, PacBio, WGS and RNA-Seq data and first draft of the manuscript was done by MZ. Hi-C data was generated by JL and analysis was done by MJ. PacBio and MinION data was generated by HF. All authors read and approved the final manuscript.
