# Supplementary material for: Linked-read based analysis of the medulloblastoma genome
Source: Front Oncol. 2023 Jul 28;13:1221611. doi: 10.3389/fonc.2023.1221611 (PMC10419201; doi:10.3389/fonc.2023.1221611)
Supplement: Supplementary file 1 [file DataSheet_1.pdf]

Linked-read based analysis of the Medulloblastoma Genome

Melissa Zwaig[1], Michael J Johnston[2], John JY Lee[4,5], Hamza Farooq[6], Marco Gallo[2], Nada Jabado[3], Michael D. Taylor[7,8*], Jiannis Ragoussis[1*]

[1] Victor Phillip Dahdaleh Institute of Genomic Medicine and Department of Human Genetics, McGill University, Montreal, Canada
[2] Alberta Children's Hospital Research Institute, Arnie Charbonneau Cancer Institute, and Department of Biochemistry and Molecular Biology, Cumming School of Medicine, University of Calgary, Calgary, AB T2N 4N1, Canada
[3] Department of Human Genetics, Department of Pediatrics, and The Research Institute of the McGill University Health Centre, Montreal, Canada
[4] Department of Pathology and Center for Cancer Research, Massachusetts General Hospital and Harvard Medical School, Boston, MA 02114, USA
[5] Broad Institute of Harvard and MIT, Cambridge, MA 02142, USA
[6] BioBox Analytics Inc., Toronto, ON. 88 College St, Toronto, ON M5G 1L4
[7] Division of Neurosurgery, The Arthur and Sonia Labatt Brain Tumour Research Centre and the Developmental and Stem Cell Biology Program, The Hospital for Sick Children, Toronto, ON, Canada
[8] Texas Children's Cancer Center, Hematology-Oncology Section and Department of Pediatrics – Hematology/Oncology and Neurosurgery, Baylor College of Medicine

*** Correspondence:** Jiannis Ragoussis (Email: ioannis.ragoussis@mcgill.ca, Phone: +1 514-398-6508), Michael D. Taylor (Email: mdt.cns@gmail.com, Phone +1 832-824-4032)

1 Supplementary Data

Supplementary_data.xlsx contains the following tables:

- Additional Table 1 – Extraction methods and QC metrics for all 10X-LR and TELL-Seq datasets
- Additional Table 2 – Manually validated somatic structural variant calls made across callers and the 10X-LR, WGS, ONT and PacBio datasets
- Additional Table 3 – Validation of point mutations in 10X-LR, WGS and RNA-Seq data
- Additional Table 4 – All structural variant calls across all linked-read technologies and callers

2 Supplementary Figures

Supplemental Figure 1. Detection of novel variants in G4 medulloblastomas. Circos plot for 10X-LR datasets showing (**a**) a complex event involving chromosomes 2 and 16 with a breakpoint in IDH1 in MDT-AP-2878. Outer circle shows allele frequency, as calculated by TitanCNA, were colour indicates the type of copy number change relative to the normal sample. Inner circle shows manually confirmed somatic SVs detected by 10X-LR and/or WGS and/or ONT and/or PacBio, colour indicates the type of SV. 10X-LR data supporting (**b**) an SV with a breakpoint downstream of IDH1 in MDT-AP-2878, (**c**) the amplification of TERT in MDT-AP-2940, visualization of the

barcode overlap shown as heat maps in Loupe. Axes represent genomic regions and the colour of the points represents the number of barcodes that map to both of these regions. (**d**) TERT promoter mutation in MDT-AP-2130.

Supplemental Figure 2. Validation of ecDNA using Hi-C data. Hi-C interaction matrix showing that two regions of chromosome 2 (15Mb and 42Mb) interact with the entirety of chromosome 2 indicating oncogenic amplification via ecDNA

Supplemental Figure 3. Copy number changes across G4 medulloblastomas. Copy number profiles of all G4 medulloblastomas across chromosome 17 (a) and throughout the whole genome (b), allele frequency calculated by TitanCNA and plotted using KaryotypeR in R.

Supplemental Figure 4. Detection of structural variants in G3 and SHH medulloblastomas. (**a**) 10X-LR data supporting an interchromosomal SV between chromosomes 3 and 14 in MDT-AP-3724, visualization of the barcode overlap shown as heat maps in Loupe. Axes represent genomic regions and the colour of the points represents the number of barcodes that map to both of these regions. (**b**) Copy number profile for MDT-AP-3724, calculated and plotted with TitanCNA. (**c**) 10X-LR data supporting an interchromosomal SV between chromosomes 7 and 18 in MDT-AP-3862, visualization of the barcode overlap shown as heat maps in Loupe. Axes represent genomic regions and the colour of the points represents the number of barcodes that map to both of these regions. (**d**) Copy number profile for MDT-AP-3862, calculated and plotted with TitanCNA. 10X-LR data supporting a germline interchromosomal structural variant in MDT-AP-4037 between chromosomes 2 and 5 in (**e**) the tumor and (**f**) the blood, visualization of the barcode overlap shown as heat maps in Loupe. Axes represent genomic regions and the colour of the points represents the number of barcodes that map to both of these regions.

Supplemental Figure 5. Comparison of 10x Genomics and Universal Sequencing Technologies' linked-read protocols. Coverage profiles for chromosome 1 using WGS, 10X-LR and TELL-Seq data, coverage calculated using BVAtools depthofcoverage and plotted using the karyoploteR.

Supplemental Figure 6. Detection of copy number changes and structural variants for all medulloblastomas. Circos plots for 10X-LR datasets showing the copy number profile and manually confirmed structural variants of 21 G4 MBs, 2 G3 MBs and 2 SHH MBs. Outer circle shows allele frequency, as calculated by TitanCNA, were colour indicates the type of copy number change relative to the normal sample. Inner circle shows manually confirmed somatic SVs detected by 10X-LR and/or WGS and/or ONT and/or PacBio, colour indicates the type of SV.

Supplemental Table 1. Sample information and dataset availability

Sample	Age	Sex	Diagnosis	Linked-reads	Illumina WGS	RNA-Seq	PacBio	Nanopore	TELL-Seq
MDT-AP-0074	3.29	M	Group 4	Tumor-Normal	Tumor-Normal	Tumor only	Tumor-Normal		
MDT-AP-1206	1	F	Group 4	Tumor-Normal	Tumor-Normal	Tumor only			Tumor only
MDT-AP-1209	8	M	Group 4	Tumor-Normal	Tumor-Normal	Tumor only			
MDT-AP-1367	8.13	M	Group 4	Tumor-Normal	Tumor-Normal	Tumor only	Tumor-Normal	Tumor-Normal	
MDT-AP-1405	8.79	M	Group 4	Tumor-Normal	Tumor-Normal		Tumor-Normal	Tumor-Normal	
MDT-AP-2075	7	F	Group 4	Tumor-Normal	Tumor-Normal	Tumor only	Tumor-Normal		
MDT-AP-2078	5.4	F	Group 4	Tumor-Normal					
MDT-AP-2130	9	M	Group 4	Tumor-Normal	Tumor-Normal	Tumor only			
MDT-AP-2151	13.47	M	Group 4	Tumor-Normal	Tumor-Normal	Tumor only			
MDT-AP-2407	6	M	Group 4	Tumor-Normal	Tumor-Normal	Tumor only			
MDT-AP-2638	NA	M	Group 4	Tumor-Normal		Tumor only			
MDT-AP-2673	10.1	F	Group 4	Tumor-Normal	Tumor-Normal	Tumor only	Tumor-Normal	Tumor only	Tumor only
MDT-AP-2849	10	F	Group 4	Tumor-Normal	Tumor-Normal	Tumor only			
MDT-AP-2857	12	M	Group 4	Tumor-Normal		Tumor only			
MDT-AP-2859	17	M	Group 4	Tumor-Normal	Tumor-Normal	Tumor only			
MDT-AP-2878	9	M	Group 4	Tumor-Normal	Tumor-Normal				Tumor only
MDT-AP-2940	22	F	Group 4	Tumor-Normal					Tumor only
MDT-AP-3670	7	M	Group 4	Tumor-Normal					
MDT-AP-3716	15	F	Group 4	Tumor-Normal					
MDT-AP-3743	6	M	Group 4	Tumor-Normal					
MDT-AP-3769	3	F	Group 4	Tumor-Normal					
MDT-AP-3667	11.3	F	Group 3	Tumor-Normal					
MDT-AP-4037	9.5	M	Group 3	Tumor-Normal					
MDT-AP-3724	14	F	SHH	Tumor-Normal					
MDT-AP-3862	2	M	SHH	Tumor-Normal					

Tumor-Normal indicates that datasets for both the tumor and corresponding blood or normal tissue were generated with the technology
Tumor only indicates that only a dataset from the tumor was generated with that technology

Supplemental Figure 1

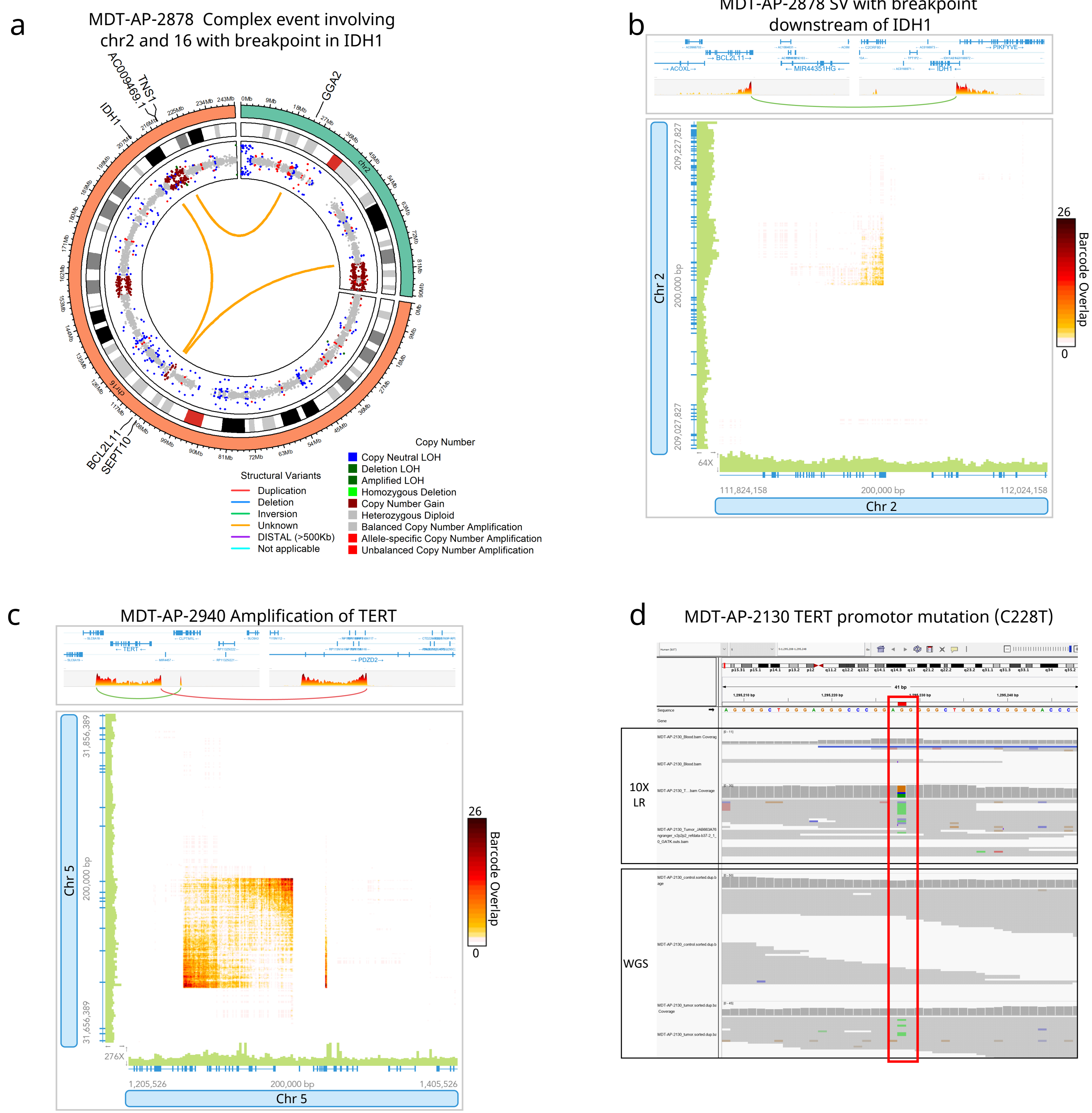

Supplemental Figure 2

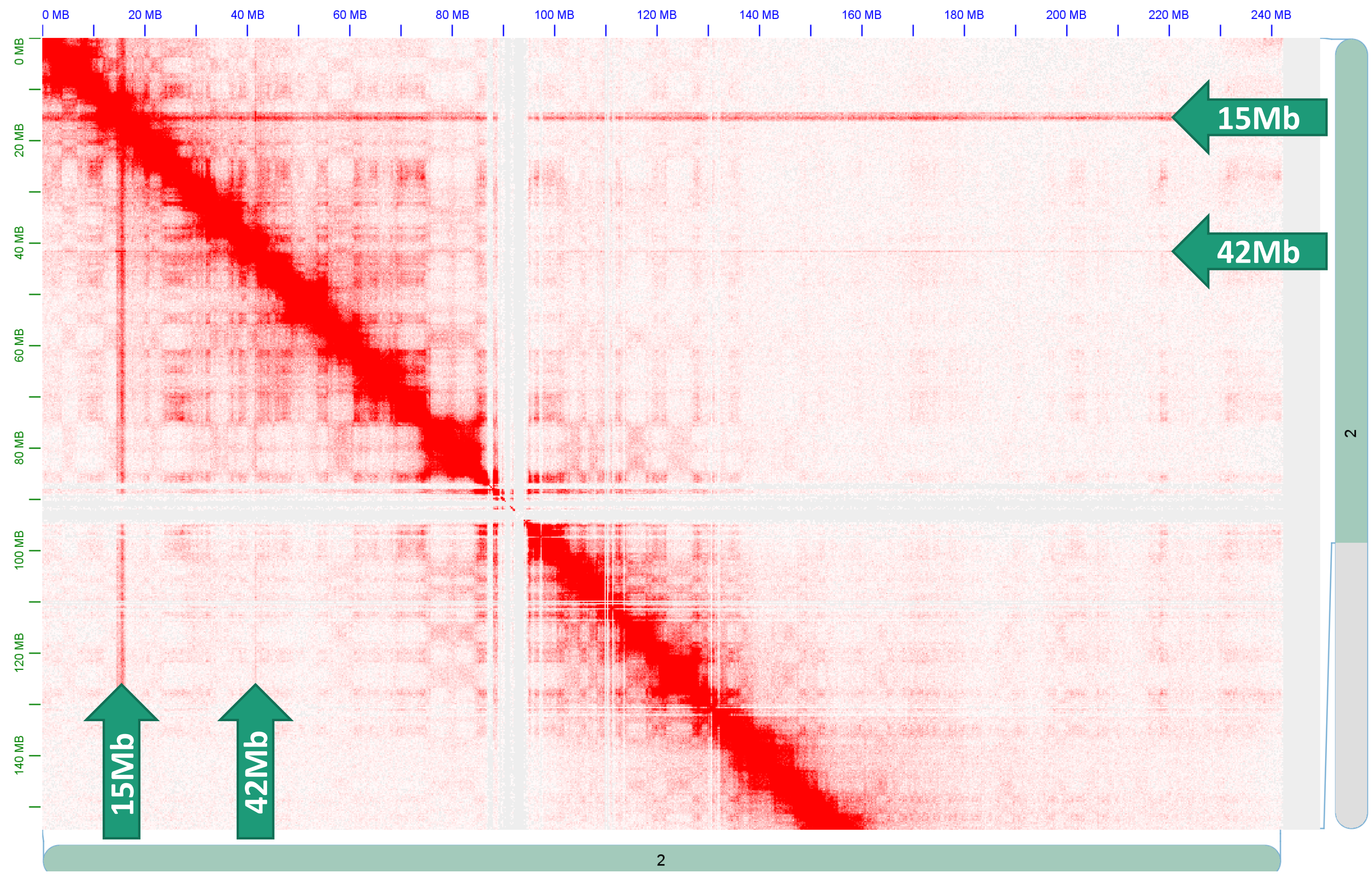

Supplemental Figure 3

a

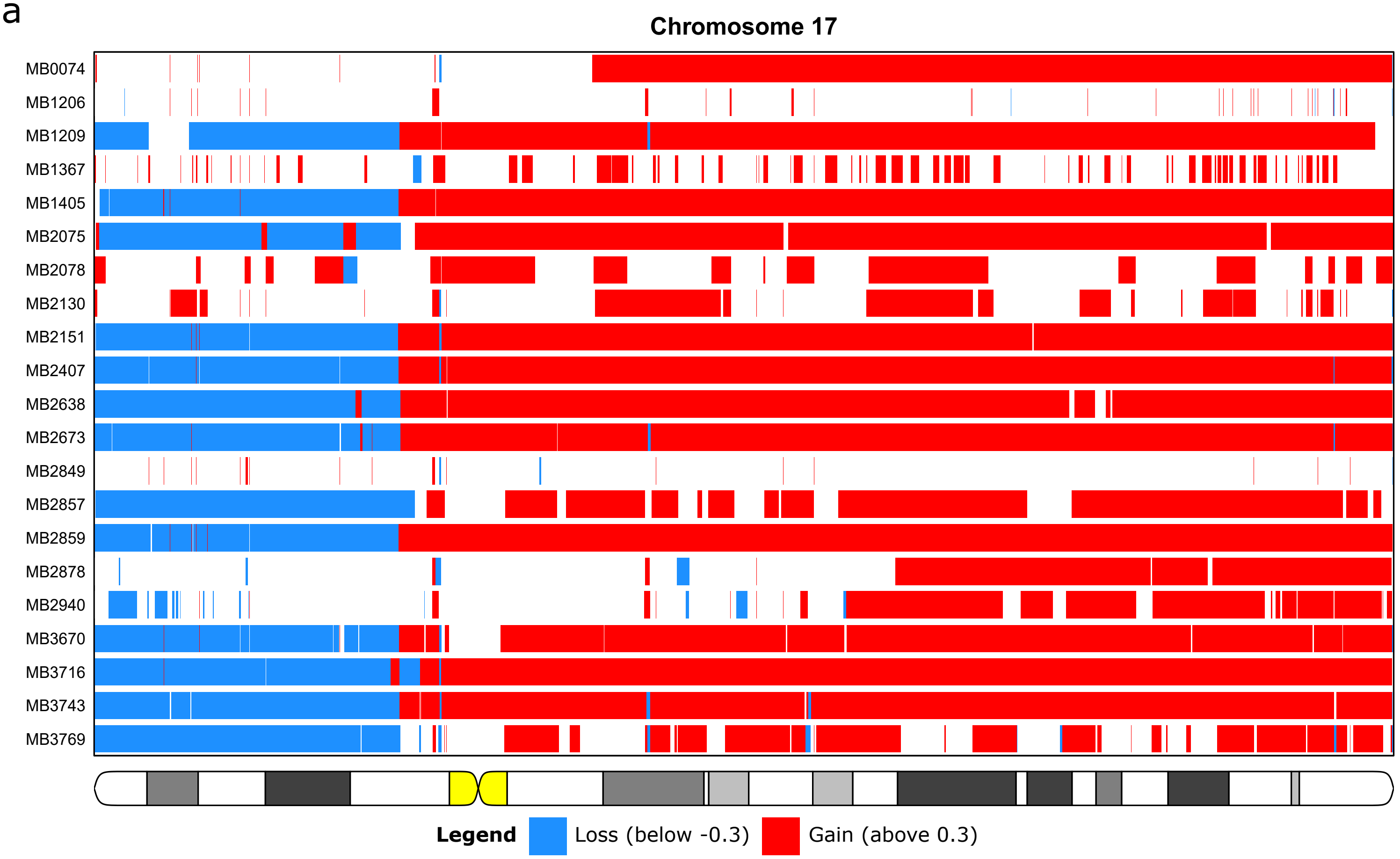

b

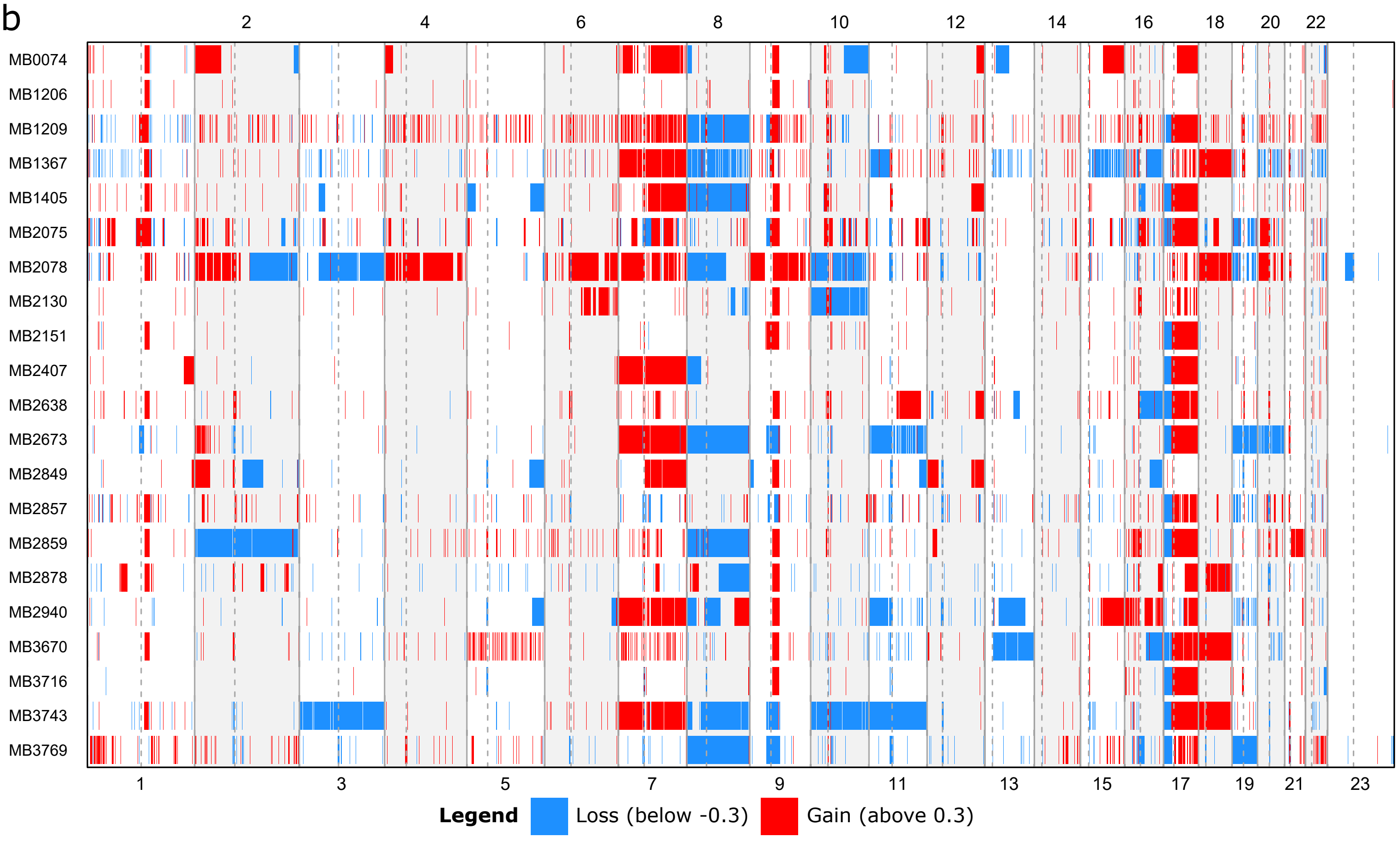

Supplemental Figure 4

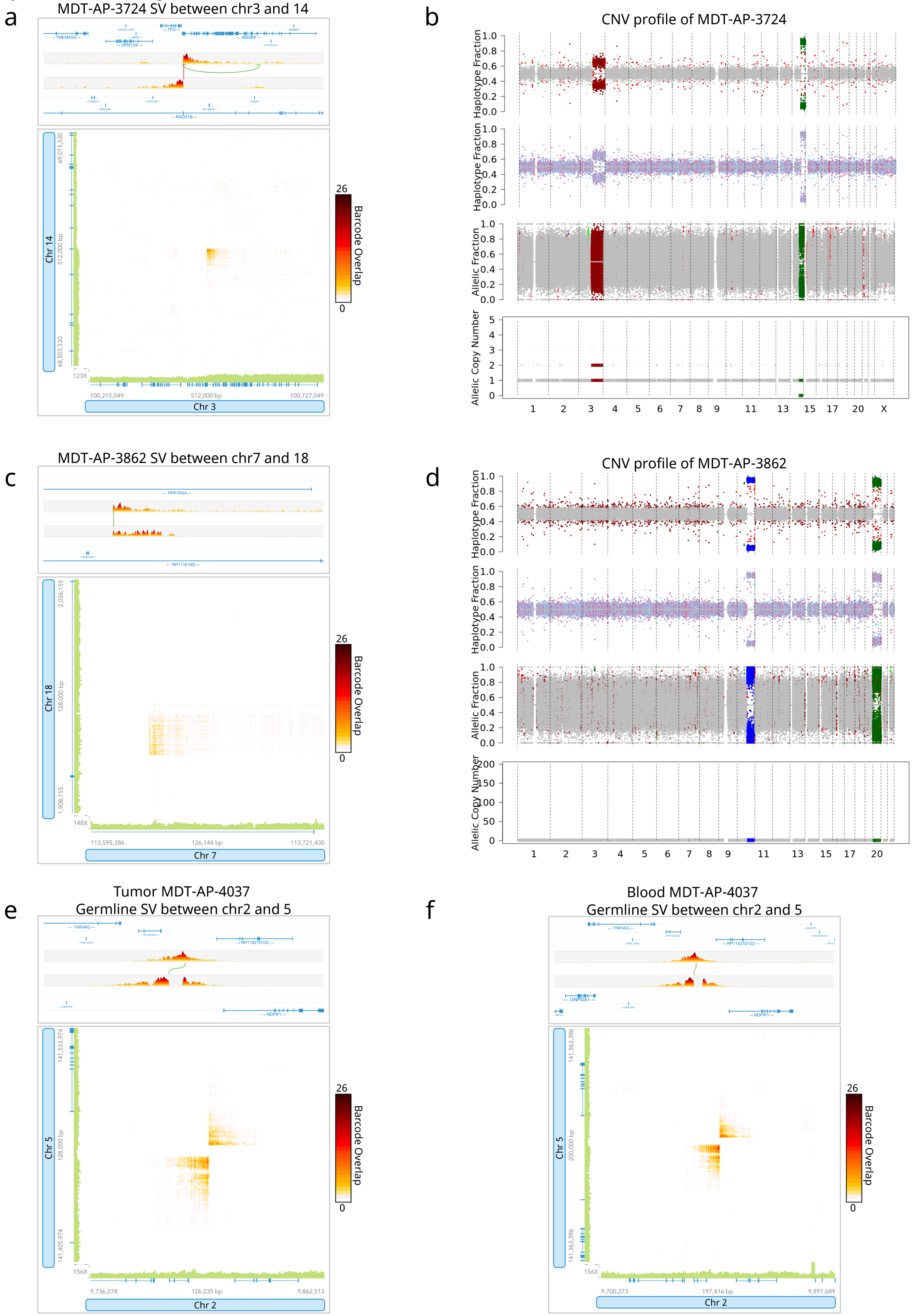

Supplemental Table 2. Comparison of QC metrics between 10X-LR and TELL-Seq

	10X Genomics								TELL-Seq							
Sample	Extraction and Size selection method	SNPs phased	Longest phase block (Mb)	Mean molecule length (kb)	Mean depth	Num large SV calls	Num short deletions	Corrected loaded Mass (ng)	Extraction and Size selection method	SNPs phased	Longest phase block (Mb)	Mean molecule length (kb)	Mean depth	Num large SV calls	Num short deletions	Corrected loaded Mass (ng)
MDT-AP-1206	PurGene	99.12%	2.78Mb	23.4kb	37.84X	1180	4132	1.08	Same extraction as 10X-LR	95.79%	1.91Mb	19.94kb	34.37X	1792	3649	0.145
MDT-AP-2673	Circulomics	99.42%	32.03Mb	93.89kb	37.74X	231	4918	1.553	Same extraction as 10X-LR	97.78%	4.25Mb	47.96kb	35.48X	292	3584	0.289
MDT-AP-2878	PurGene and SRE Circulomics	97.74%	4.00Mb	34.44kb	30.45X	674	8232	0.417	Same extraction as 10X-LR	96.77%	3.05Mb	35.5kb	34.28X	587	3718	0.255
MDT-AP-2940	PurGene	99.64%	9.74Mb	30.98kb	99.13X	196	3925	1.669	Same extraction as 10X-LR	95.51%	5.95Mb	30.46kb	36.32X	971	4510	0.712

Supplemental Figure 5

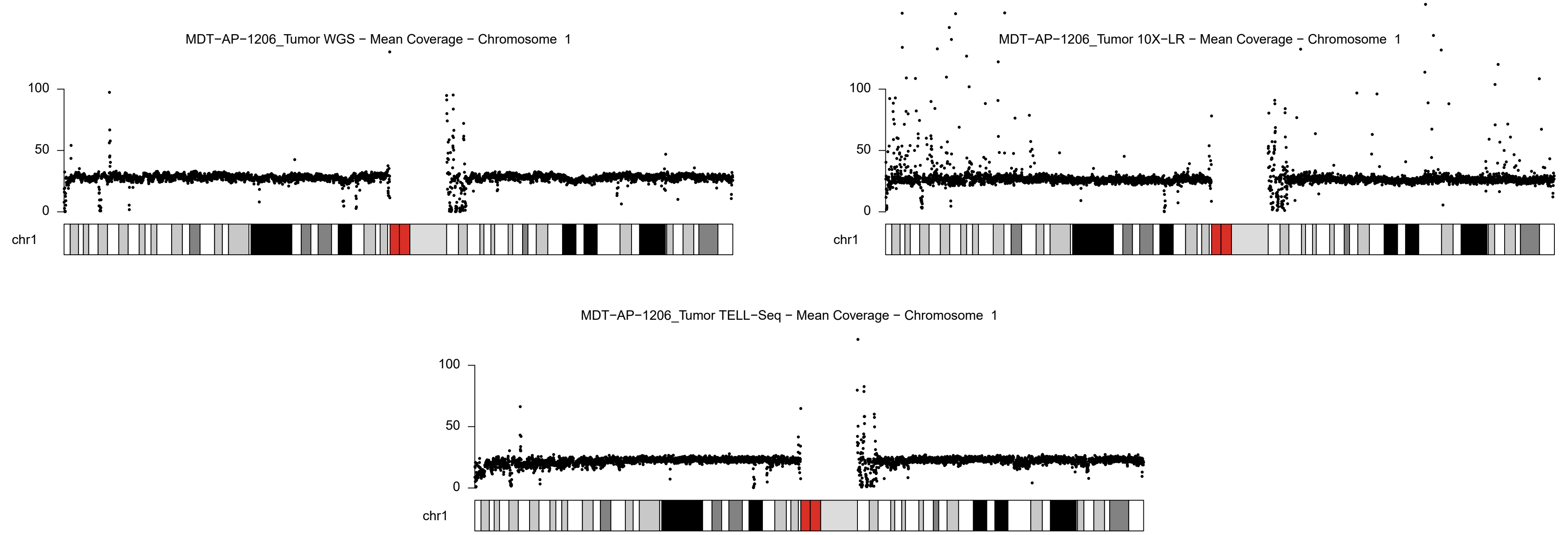

Supplemental Figure 6

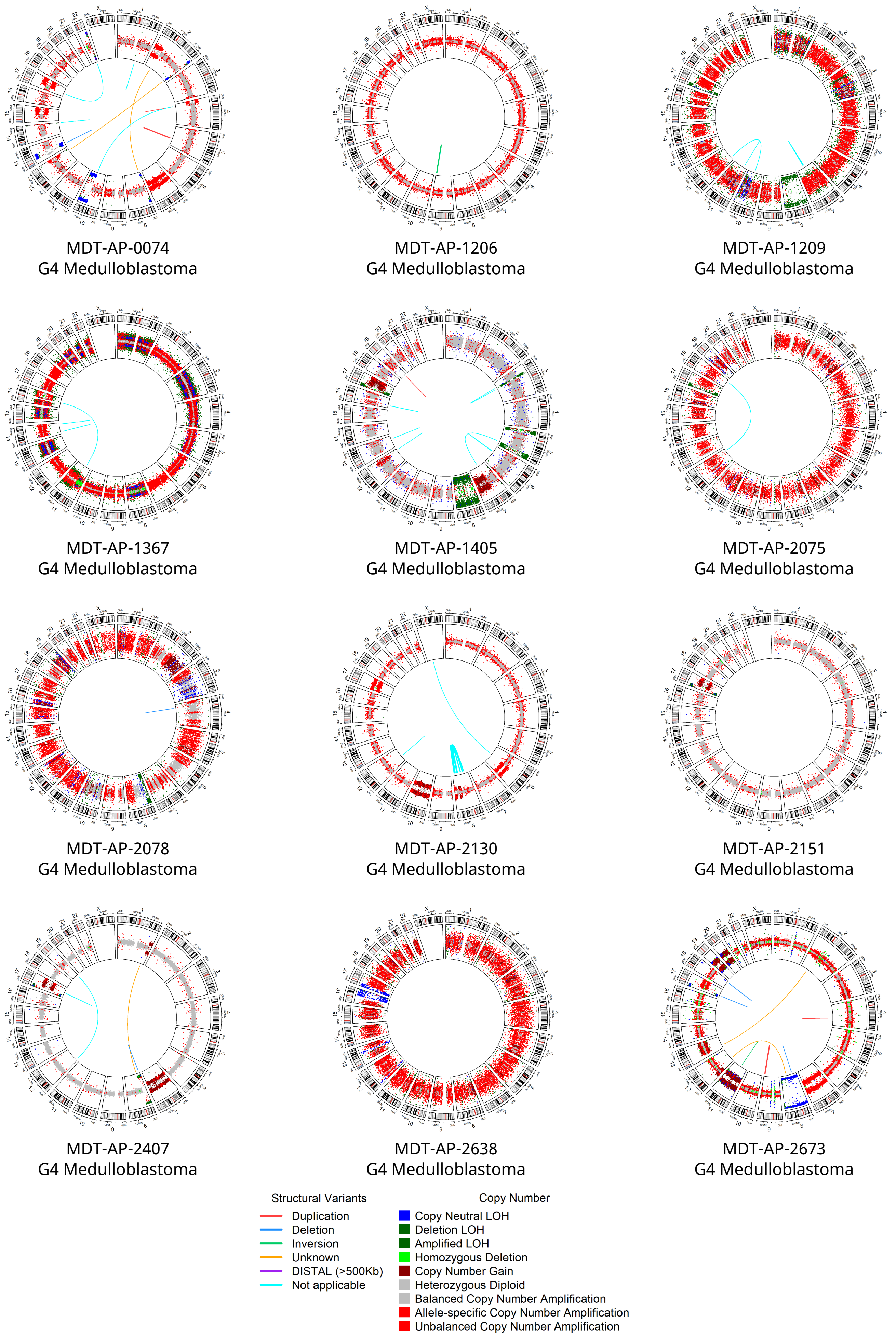

Supplemental Figure 6 Cont.

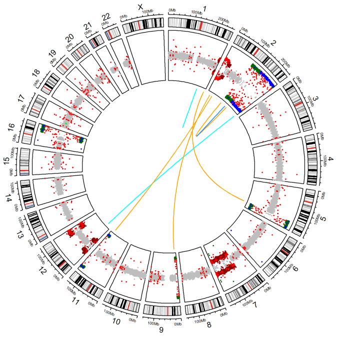

MDT-AP-2849
G4 Medulloblastoma

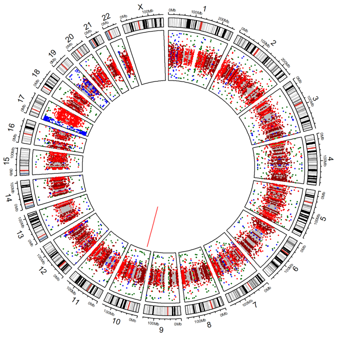

MDT-AP-2857
G4 Medulloblastoma

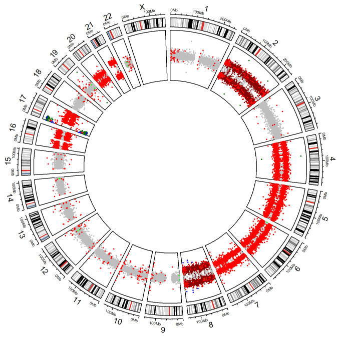

MDT-AP-2859
G4 Medulloblastoma

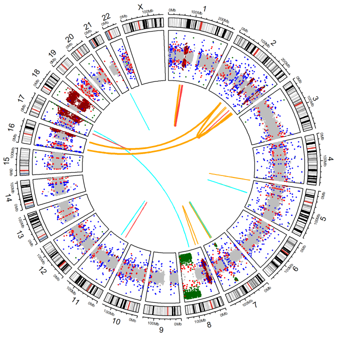

MDT-AP-2878
G4 Medulloblastoma

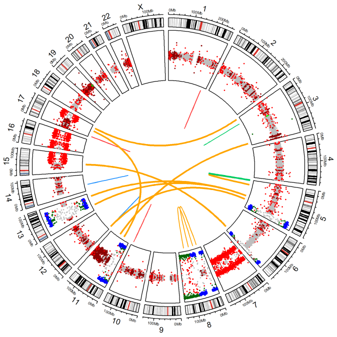

MDT-AP-2940
G4 Medulloblastoma

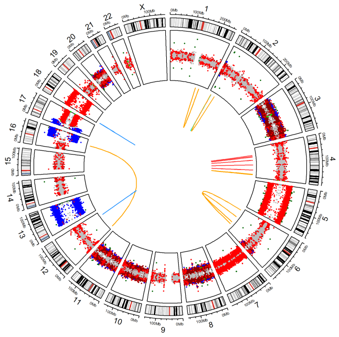

MDT-AP-3670
G4 Medulloblastoma

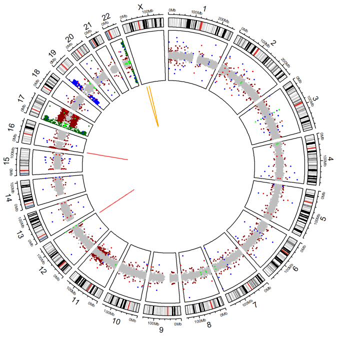

MDT-AP-3716
G4 Medulloblastoma

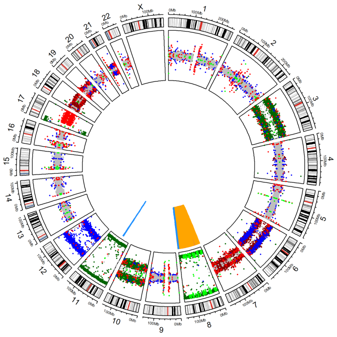

MDT-AP-3743
G4 Medulloblastoma

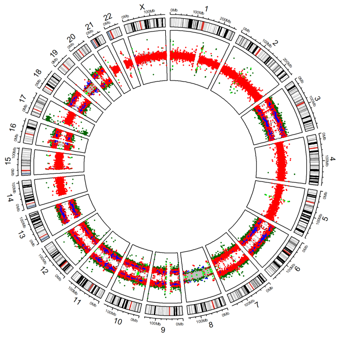

MDT-AP-3769
G4 Medulloblastoma

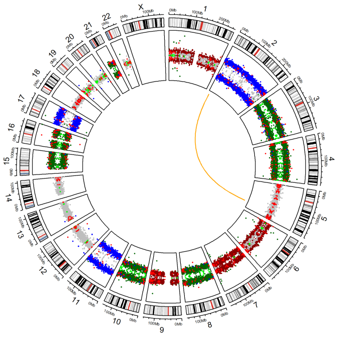

MDT-AP-4037
G3 Medulloblastoma

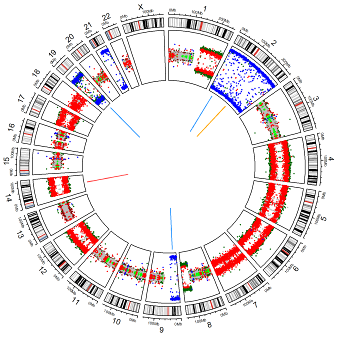

MDT-AP-3667
G3 Medulloblastoma

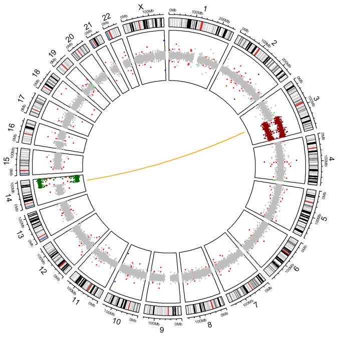

MDT-AP-3724
SHH Medulloblastoma

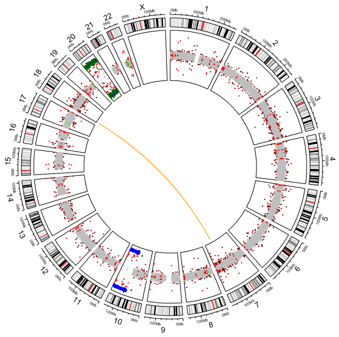

MDT-AP-3862
SHH Medulloblastoma

Structural Variants
- Duplication
- Deletion
- Inversion
- Unknown
- DISTAL (>500Kb)
- Not applicable

Copy Number
- Copy Neutral LOH
- Deletion LOH
- Amplified LOH
- Homozygous Deletion
- Copy Number Gain
- Heterozygous Diploid
- Balanced Copy Number Amplification
- Allele-specific Copy Number Amplification
- Unbalanced Copy Number Amplification
